# (*E*)-4-Amino-*N*′-(5-bromo-2-hy­droxy­benzyl­idene)benzohydrazide mono­hydrate

**DOI:** 10.1107/S1600536812025950

**Published:** 2012-06-16

**Authors:** Hadi Kargar, Reza Kia, Muhammad Nawaz Tahir

**Affiliations:** a Department of Chemistry, Payame Noor University, PO BOX 19395-3697 Tehran, I. R. of IRAN; b Department of Chemistry, Science and Research Branch, Islamic Azad University, Tehran, Iran; c Department of Physics, University of Sargodha, Punjab, Pakistan

## Abstract

In t the title compound, C_14_H_12_BrN_3_O_2_. H_2_O, the conformation of the C=N double bond in the hydrazide Schiff base mol­ecule is *E*. The dihedral angle between the benzene rings is 48.01 (11) °. An intra­molecular O—H⋯N hydrogen bond makes an *S*(6) ring motif. In the crystal, mol­ecules are linked through N—H⋯O (bifurcated acceptor) and O—H⋯O hydrogen bonds, forming two-dimensional networks lying parallel to (100).

## Related literature

For the coordination chemistry of Schiff base and hydrazone derivatives, see: Kucukguzel *et al.* (2006 ▶); Karthikeyan *et al.* (2006 ▶). For 4-amino­benzohydrazide-derived Schiff base structures, see: Xu (2012 ▶); Shi & Li (2012 ▶); Bakir & Green (2002 ▶). For standard bond lengths, see: Allen *et al.* (1987 ▶). For hydrogen-bond motifs, see: Bernstein *et al.* (1995 ▶).
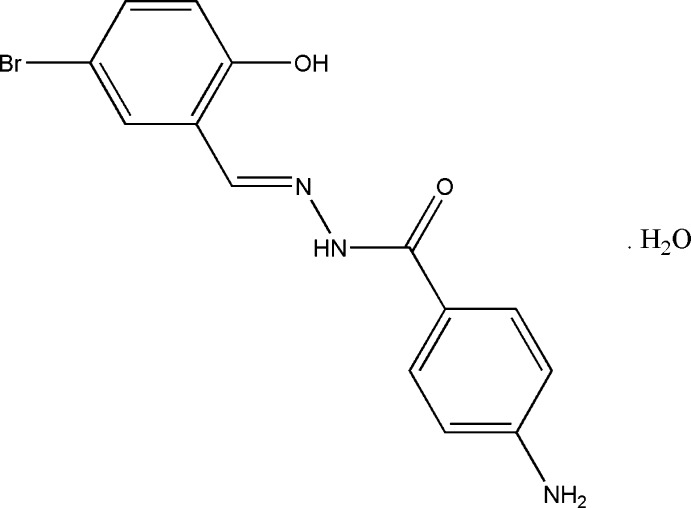


## Experimental

### 

#### Crystal data

C_14_H_12_BrN_3_O_2_·H_2_O
*M* *_r_* = 352.19
Monoclinic, 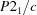

*a* = 15.8435 (11) Å
*b* = 7.1718 (6) Å
*c* = 12.6462 (8) Å
β = 101.391 (3)°
*V* = 1408.64 (18) Å^3^
*Z* = 4
Mo *K*α radiation
μ = 2.93 mm^−1^
*T* = 291 K
0.32 × 0.26 × 0.22 mm

#### Data collection

Bruker SMART APEXII CCD area-detector diffractometer
Absorption correction: multi-scan (*SADABS*; Bruker, 2005 ▶) *T*_min_ = 0.454, *T*_max_ = 0.565
11798 measured reflections
3138 independent reflections
2543 reflections with *I* > 2σ(*I*)
*R*_int_ = 0.031

#### Refinement

*R*[*F* ^2^ > 2σ(*F* ^2^)] = 0.030
*wR*(*F* ^2^) = 0.077
*S* = 1.04
3138 reflections
190 parameters
H-atom parameters constrained
Δρ_max_ = 0.27 e Å^−3^
Δρ_min_ = −0.49 e Å^−3^

### 

Data collection: *APEX2* (Bruker, 2005 ▶); cell refinement: *SAINT* (Bruker, 2005 ▶); data reduction: *SAINT*; program(s) used to solve structure: *SHELXS97* (Sheldrick, 2008 ▶); program(s) used to refine structure: *SHELXL97* (Sheldrick, 2008 ▶); molecular graphics: *SHELXTL* (Sheldrick, 2008 ▶); software used to prepare material for publication: *SHELXTL* and *PLATON* (Spek, 2009 ▶).

## Supplementary Material

Crystal structure: contains datablock(s) global, I. DOI: 10.1107/S1600536812025950/su2450sup1.cif

Structure factors: contains datablock(s) I. DOI: 10.1107/S1600536812025950/su2450Isup2.hkl

Supplementary material file. DOI: 10.1107/S1600536812025950/su2450Isup3.cml

Additional supplementary materials:  crystallographic information; 3D view; checkCIF report

## Figures and Tables

**Table 1:** Hydrogen-bond geometry (Å, °)

*D*—H⋯*A*	*D*—H	H⋯*A*	*D*⋯*A*	*D*—H⋯*A*
O2—H2*O*⋯N3	0.93	1.84	2.660 (2)	145
N2—H2*N*⋯O1*W* ^i^	0.90	1.93	2.823 (2)	171
N1—H2*N*1⋯O1*W* ^ii^	0.89	2.57	3.276 (3)	137
O1*W*—H2*W*1⋯O1	0.90	1.77	2.653 (2)	167

Symmetry codes: (i) 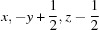; (ii) 
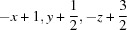
.

**Fig. 1.**
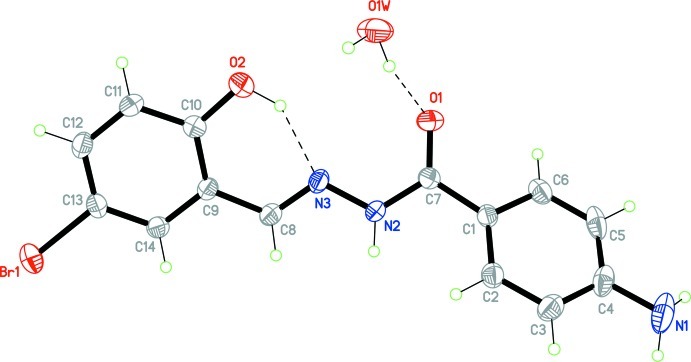
A view of the molecular structure of the title compound, showing 40% probability displacement ellipsoids and the atom numbering. The dashed lines show the intramolecular N-H···O hydrogen bonds (see Table 1 for details).

**Fig. 2.**
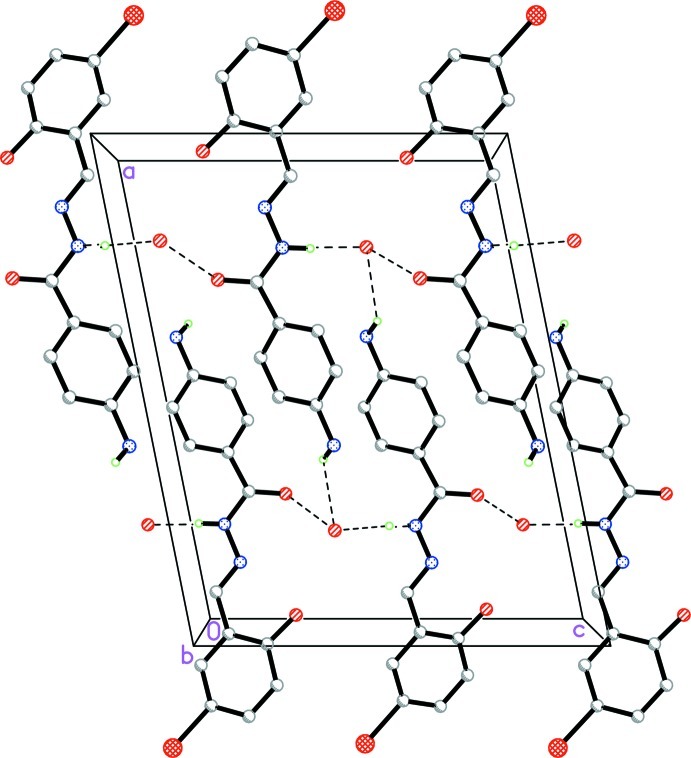
A view along the b axis of crystal packing of the title compound, showing the linking of molecules through N—H···O and O—H···O hydrogen bonds (dashed lines; see Table 1 for details; only the NH H atom is shown).

## supplementary crystallographic information

### Comment

Schiff bases are one of the most prevalent mixed-donor ligands in the field of coordination chemistry. They play an important role in the development of coordination chemistry related to catalysis and magnetism, and supramolecular architectures (Karthikeyan *et al.*, 2006; Kucukguzel *et al.*, 2006). Structures of Schiff bases derived from substituted 4-aminobenzohydrazide have been reported earlier (Xu, 2012; Shi & Li, 2012; Bakir & Green, 2002). In order to explore the structure of the new Schiff base derivatives, the title compound was prepared and characterized crystallographically.

The asymmetric unit of the title compound, Fig. 1, comprises a molecule of the title hydrazide Schiff base and a water molecule of crystallization. The hydrazide Schiff base shows an *E* conformation around the C═N double bond. The bond lengths (Allen *et al.*, 1987) and angles are within normal ranges and are comparable to those reported for related structures (Xu, 2012; Shi & Li, 2012; Bakir & Green, 2002). An intramolecular O—H···N hydrogen bond makes an *S(6)* ring motif (Table 1; Bernstein *et al.*, 1995). The dihedral angle between the benzene rings is 48.01 (11)Å.

In the crystal, molecules are linked through N—H···O [bifurcated acceptor] and O—H···O hydrogen bonds forming two-dimensional networks lying parallel to (100) [Table 1 and Fig. 2].

### Experimental

The title compound was synthesized by adding 1 mmol of methyl 4-aminobenzoate to a solution of 5-Bromosalicylaldehyde (1 mmol) in methanol (30 ml). The mixture was refluxed with stirring for 50 min and after cooling to room temperature a light-yellow precipitate was filtered and washed with diethylether and dried in air. Light-yellow prismatic crystals of the title compound, suitable for *X*-ray structure analysis, were recrystallized from ethanol by slow evaporation of the solvents at room temperature over several days.

### Refinement

The N- and O-bound H-atoms were located in a difference Fourier map and were constrained to ride on the parent atom with U_iso_(H) = 1.2U_eq_(N) and 1.5U_eq_(O). The C-bound H atoms were included in calculated positions and treated by the riding model approximation: C—H = 0.93 Å with U_iso_ (H) = 1.2U_eq_(C).

### Crystal data

**Table:**

C_14_H_12_BrN_3_O_2_·H_2_O	*F*(000) = 712
*M**_r_* = 352.19	*D*_x_ = 1.661 Mg m^−^^3^
Monoclinic, *P*2_1_/*c*	Mo *K*α radiation, λ = 0.71073 Å
Hall symbol: -P 2ybc	Cell parameters from 2225 reflections
*a* = 15.8435 (11) Å	θ = 2.5–27.5°
*b* = 7.1718 (6) Å	µ = 2.93 mm^−^^1^
*c* = 12.6462 (8) Å	*T* = 291 K
β = 101.391 (3)°	Prism, white
*V* = 1408.64 (18) Å^3^	0.32 × 0.26 × 0.22 mm
*Z* = 4	

### Data collection

**Table:**

Bruker SMART APEXII CCD area-detector diffractometer	3138 independent reflections
Radiation source: fine-focus sealed tube	2543 reflections with *I* > 2σ(*I*)
Graphite monochromator	*R*_int_ = 0.031
φ and ω scans	θ_max_ = 27.3°, θ_min_ = 2.6°
Absorption correction: multi-scan (*SADABS*; Bruker, 2005)	*h* = −20→20
*T*_min_ = 0.454, *T*_max_ = 0.565	*k* = −9→8
11798 measured reflections	*l* = −9→16

### Refinement

**Table:**

Refinement on *F*^2^	Primary atom site location: structure-invariant direct methods
Least-squares matrix: full	Secondary atom site location: difference Fourier map
*R*[*F*^2^ > 2σ(*F*^2^)] = 0.030	Hydrogen site location: inferred from neighbouring sites
*wR*(*F*^2^) = 0.077	H-atom parameters constrained
*S* = 1.04	*w* = 1/[σ^2^(*F*_o_^2^) + (0.0361*P*)^2^ + 0.4505*P*] where *P* = (*F*_o_^2^ + 2*F*_c_^2^)/3
3138 reflections	(Δ/σ)_max_ = 0.001
190 parameters	Δρ_max_ = 0.27 e Å^−^^3^
0 restraints	Δρ_min_ = −0.49 e Å^−^^3^

### Special details

**Table:**

Geometry. All esds (except the esd in the dihedral angle between two l.s. planes) are estimated using the full covariance matrix. The cell esds are taken into account individually in the estimation of esds in distances, angles and torsion angles; correlations between esds in cell parameters are only used when they are defined by crystal symmetry. An approximate (isotropic) treatment of cell esds is used for estimating esds involving l.s. planes.
Refinement. Refinement of F^2^ against ALL reflections. The weighted R-factor wR and goodness of fit S are based on F^2^, conventional R-factors R are based on F, with F set to zero for negative F^2^. The threshold expression of F^2^ > 2sigma(F^2^) is used only for calculating R-factors(gt) etc. and is not relevant to the choice of reflections for refinement. R-factors based on F^2^ are statistically about twice as large as those based on F, and R- factors based on ALL data will be even larger.

### Fractional atomic coordinates and isotropic or equivalent isotropic displacement parameters (Å^2^)

**Table:**

	*x*	*y*	*z*	*U*_iso_*/*U*_eq_	
Br1	−0.256908 (14)	0.19559 (3)	0.351278 (19)	0.04528 (10)	
O1	0.27754 (10)	0.3064 (2)	0.77430 (12)	0.0463 (4)	
O2	0.02746 (10)	0.1315 (3)	0.74256 (12)	0.0490 (4)	
H2O	0.0786	0.1768	0.7273	0.073*	
O1W	0.20712 (12)	0.0602 (3)	0.88744 (12)	0.0555 (5)	
H1W1	0.1801	−0.0294	0.8452	0.083*	
H2W1	0.2246	0.1388	0.8402	0.083*	
N1	0.61249 (13)	0.3236 (4)	0.5692 (2)	0.0675 (7)	
H1N1	0.6101	0.3120	0.4985	0.081*	
H2N1	0.6429	0.4174	0.6041	0.081*	
N2	0.21269 (10)	0.3032 (2)	0.59806 (14)	0.0322 (4)	
H2N	0.2133	0.3342	0.5294	0.039*	
N3	0.13313 (11)	0.2630 (2)	0.62093 (15)	0.0321 (4)	
C1	0.36621 (12)	0.3254 (3)	0.64417 (16)	0.0297 (4)	
C2	0.38195 (13)	0.2378 (3)	0.55174 (17)	0.0344 (5)	
H2	0.3372	0.1774	0.5059	0.041*	
C3	0.46277 (15)	0.2394 (3)	0.5272 (2)	0.0425 (5)	
H3	0.4721	0.1783	0.4657	0.051*	
C4	0.53040 (14)	0.3309 (3)	0.5931 (2)	0.0428 (6)	
C5	0.51473 (13)	0.4213 (4)	0.6841 (2)	0.0475 (6)	
H5	0.5592	0.4855	0.7283	0.057*	
C6	0.43430 (13)	0.4174 (3)	0.70993 (17)	0.0384 (5)	
H6	0.4254	0.4771	0.7721	0.046*	
C7	0.28256 (13)	0.3116 (3)	0.67866 (16)	0.0304 (4)	
C8	0.06943 (13)	0.2690 (3)	0.54186 (17)	0.0321 (4)	
H7	0.0781	0.3071	0.4745	0.039*	
C9	−0.01679 (12)	0.2173 (3)	0.55511 (16)	0.0284 (4)	
C10	−0.03425 (13)	0.1483 (3)	0.65254 (17)	0.0328 (4)	
C11	−0.11712 (13)	0.0932 (3)	0.65825 (17)	0.0369 (5)	
H11	−0.1282	0.0459	0.7226	0.044*	
C12	−0.18330 (12)	0.1076 (3)	0.56950 (17)	0.0352 (5)	
H12	−0.2389	0.0715	0.5740	0.042*	
C13	−0.16619 (12)	0.1761 (3)	0.47410 (17)	0.0317 (4)	
C14	−0.08416 (13)	0.2293 (3)	0.46589 (17)	0.0304 (4)	
H14	−0.0737	0.2735	0.4005	0.037*	

### Atomic displacement parameters (Å^2^)

**Table:**

	*U*^11^	*U*^22^	*U*^33^	*U*^12^	*U*^13^	*U*^23^
Br1	0.03422 (13)	0.04959 (16)	0.04628 (16)	−0.00074 (10)	−0.00607 (10)	−0.00069 (10)
O1	0.0456 (9)	0.0669 (12)	0.0280 (8)	−0.0112 (8)	0.0110 (7)	−0.0009 (7)
O2	0.0392 (8)	0.0719 (12)	0.0331 (8)	−0.0078 (8)	0.0005 (6)	0.0088 (8)
O1W	0.0755 (12)	0.0603 (12)	0.0319 (9)	−0.0168 (9)	0.0136 (8)	−0.0011 (8)
N1	0.0289 (10)	0.0796 (18)	0.0962 (19)	0.0066 (10)	0.0174 (11)	0.0289 (14)
N2	0.0246 (8)	0.0455 (11)	0.0279 (9)	−0.0050 (7)	0.0084 (7)	0.0004 (7)
N3	0.0251 (8)	0.0364 (9)	0.0369 (10)	−0.0046 (7)	0.0109 (7)	−0.0024 (7)
C1	0.0260 (9)	0.0326 (11)	0.0294 (10)	−0.0018 (8)	0.0029 (8)	0.0037 (8)
C2	0.0288 (10)	0.0368 (12)	0.0368 (12)	−0.0025 (8)	0.0047 (9)	−0.0015 (9)
C3	0.0394 (12)	0.0462 (13)	0.0447 (14)	0.0069 (10)	0.0156 (10)	0.0046 (10)
C4	0.0267 (10)	0.0430 (13)	0.0593 (15)	0.0052 (9)	0.0101 (10)	0.0219 (11)
C5	0.0277 (11)	0.0473 (14)	0.0599 (16)	−0.0101 (10)	−0.0096 (10)	0.0089 (12)
C6	0.0363 (11)	0.0407 (12)	0.0347 (12)	−0.0045 (9)	−0.0014 (9)	−0.0004 (9)
C7	0.0301 (10)	0.0335 (11)	0.0274 (10)	−0.0026 (8)	0.0049 (8)	0.0004 (8)
C8	0.0294 (10)	0.0361 (11)	0.0326 (11)	−0.0005 (8)	0.0104 (8)	−0.0012 (8)
C9	0.0259 (9)	0.0288 (10)	0.0317 (11)	−0.0011 (7)	0.0086 (8)	−0.0016 (8)
C10	0.0320 (10)	0.0372 (11)	0.0287 (10)	−0.0008 (8)	0.0051 (8)	−0.0008 (8)
C11	0.0362 (11)	0.0447 (13)	0.0327 (11)	−0.0062 (10)	0.0135 (9)	0.0005 (9)
C12	0.0267 (10)	0.0376 (12)	0.0427 (12)	−0.0050 (9)	0.0105 (8)	−0.0052 (10)
C13	0.0267 (9)	0.0319 (11)	0.0347 (11)	0.0006 (8)	0.0021 (8)	−0.0060 (8)
C14	0.0312 (10)	0.0318 (11)	0.0290 (11)	−0.0002 (8)	0.0077 (8)	−0.0003 (8)

### Geometric parameters (Å, º)

**Table:**

Br1—C13	1.902 (2)	C3—C4	1.386 (4)
O1—C7	1.228 (2)	C3—H3	0.9300
O2—C10	1.352 (3)	C4—C5	1.384 (4)
O2—H2O	0.9276	C5—C6	1.377 (3)
O1W—H1W1	0.8893	C5—H5	0.9300
O1W—H2W1	0.9036	C6—H6	0.9300
N1—C4	1.393 (3)	C8—C9	1.457 (3)
N1—H1N1	0.8915	C8—H7	0.9300
N1—H2N1	0.8923	C9—C14	1.394 (3)
N2—C7	1.350 (3)	C9—C10	1.405 (3)
N2—N3	1.378 (2)	C10—C11	1.387 (3)
N2—H2N	0.8985	C11—C12	1.380 (3)
N3—C8	1.273 (3)	C11—H11	0.9300
C1—C6	1.392 (3)	C12—C13	1.378 (3)
C1—C2	1.392 (3)	C12—H12	0.9300
C1—C7	1.478 (3)	C13—C14	1.378 (3)
C2—C3	1.376 (3)	C14—H14	0.9300
C2—H2	0.9300		
			
C10—O2—H2O	108.0	C1—C6—H6	119.6
H1W1—O1W—H2W1	103.2	O1—C7—N2	122.64 (19)
C4—N1—H1N1	111.3	O1—C7—C1	121.92 (19)
C4—N1—H2N1	107.5	N2—C7—C1	115.44 (18)
H1N1—N1—H2N1	118.5	N3—C8—C9	121.11 (19)
C7—N2—N3	119.90 (17)	N3—C8—H7	119.4
C7—N2—H2N	123.7	C9—C8—H7	119.4
N3—N2—H2N	116.1	C14—C9—C10	118.67 (18)
C8—N3—N2	116.35 (17)	C14—C9—C8	118.45 (18)
C6—C1—C2	118.00 (19)	C10—C9—C8	122.82 (18)
C6—C1—C7	119.32 (19)	O2—C10—C11	117.74 (19)
C2—C1—C7	122.52 (18)	O2—C10—C9	122.36 (18)
C3—C2—C1	121.0 (2)	C11—C10—C9	119.90 (19)
C3—C2—H2	119.5	C12—C11—C10	120.8 (2)
C1—C2—H2	119.5	C12—C11—H11	119.6
C2—C3—C4	120.8 (2)	C10—C11—H11	119.6
C2—C3—H3	119.6	C13—C12—C11	119.30 (18)
C4—C3—H3	119.6	C13—C12—H12	120.3
C5—C4—C3	118.5 (2)	C11—C12—H12	120.3
C5—C4—N1	121.8 (2)	C12—C13—C14	121.08 (19)
C3—C4—N1	119.7 (3)	C12—C13—Br1	119.59 (15)
C6—C5—C4	120.9 (2)	C14—C13—Br1	119.33 (16)
C6—C5—H5	119.5	C13—C14—C9	120.29 (19)
C4—C5—H5	119.5	C13—C14—H14	119.9
C5—C6—C1	120.8 (2)	C9—C14—H14	119.9
C5—C6—H6	119.6		
			
C7—N2—N3—C8	175.72 (19)	N2—N3—C8—C9	175.98 (18)
C6—C1—C2—C3	1.2 (3)	N3—C8—C9—C14	179.08 (19)
C7—C1—C2—C3	−174.1 (2)	N3—C8—C9—C10	−3.7 (3)
C1—C2—C3—C4	−1.1 (3)	C14—C9—C10—O2	179.73 (19)
C2—C3—C4—C5	−0.2 (3)	C8—C9—C10—O2	2.5 (3)
C2—C3—C4—N1	177.1 (2)	C14—C9—C10—C11	0.3 (3)
C3—C4—C5—C6	1.3 (3)	C8—C9—C10—C11	−176.9 (2)
N1—C4—C5—C6	−176.0 (2)	O2—C10—C11—C12	179.6 (2)
C4—C5—C6—C1	−1.2 (4)	C9—C10—C11—C12	−0.9 (3)
C2—C1—C6—C5	−0.1 (3)	C10—C11—C12—C13	0.6 (3)
C7—C1—C6—C5	175.3 (2)	C11—C12—C13—C14	0.3 (3)
N3—N2—C7—O1	−8.8 (3)	C11—C12—C13—Br1	179.78 (16)
N3—N2—C7—C1	170.52 (17)	C12—C13—C14—C9	−0.9 (3)
C6—C1—C7—O1	−30.2 (3)	Br1—C13—C14—C9	179.60 (15)
C2—C1—C7—O1	145.0 (2)	C10—C9—C14—C13	0.6 (3)
C6—C1—C7—N2	150.5 (2)	C8—C9—C14—C13	177.93 (19)
C2—C1—C7—N2	−34.3 (3)		

### Hydrogen-bond geometry (Å, º)

**Table:**

*D*—H···*A*	*D*—H	H···*A*	*D*···*A*	*D*—H···*A*
O2—H2*O*···N3	0.93	1.84	2.660 (2)	145
N2—H2*N*···O1*W*^i^	0.90	1.93	2.823 (2)	171
N1—H2*N*1···O1*W*^ii^	0.89	2.57	3.276 (3)	137
O1*W*—H2*W*1···O1	0.90	1.77	2.653 (2)	167

Symmetry codes: (i) *x*, −*y*+1/2, *z*−1/2; (ii) −*x*+1, *y*+1/2, −*z*+3/2.
